# Metastatic Salivary Gland Tumors: A Single-Center Study Demonstrating the Feasibility and Potential Clinical Benefit of Molecular-Profiling-Guided Therapy

**DOI:** 10.1155/2015/614845

**Published:** 2015-09-13

**Authors:** Aron Popovtzer, Michal Sarfaty, Dror Limon, Gideon Marshack, Eli Perlow, Addie Dvir, Lior Soussan-Gutman, Salomon M. Stemmer

**Affiliations:** ^1^Institute of Oncology, Davidoff Center, Rabin Medical Center, 39 Jabotinsky Street, 49100 Petah Tikva, Israel; ^2^Sackler Faculty of Medicine, Tel Aviv University, Ramat Aviv, 69978 Tel Aviv, Israel; ^3^Radiology Department, Rabin Medical Center, 39 Jabotinsky Street, 49100 Petah Tikva, Israel; ^4^Oncotest-Teva Pharmaceutical Industries, Ltd., Hatee'na 1, 60850 Shoham, Israel

## Abstract

We evaluated the use of molecular profiling (MP) for metastatic salivary gland adenoid cystic carcinoma (SACC), for which there is no standard treatment. MP (Caris Molecular Intelligence) was performed on biopsy samples from all metastatic SACC patients attending a tertiary medical center between 2010 and 2013 (*n* = 14). Treatment was selected according to the biomarkers identified. Findings were compared with all similarly diagnosed patients treated in the same center between 1996 and 2009 (*n* = 9). For each patient, MP identified 1–13 biomarkers associated with clinical benefit for specific therapies (most commonly low/negative TS, low ERCC1). Eleven patients (79%) received MP-guided treatment (2 died prior to treatment initiation, 1 opted not to be treated), with complete response in 1, partial response (PR) in 3, and stable disease in 4. In the historical controls, 2 patients (22%) were treated (1 had PR). Median (range) progression-free survival in the first line after MP was 8.2 months (1.4–49.5+). Median (range) overall survival from diagnosis of metastatic disease was 31.3 (1.4–71.1+) versus 14.0 (1.5–116) months in the historical controls. In conclusion, MP expands treatment options and may improve clinical outcomes for metastatic SACC. In orphan diseases where randomized trials cannot be performed, MP could become a standard clinical tool.

## 1. Introduction

Salivary gland adenoid cystic carcinoma (SACC) is characterized by slow progression, although recurrences after short disease-free intervals are relatively common. Once the disease metastasizes, one-third of patients die within 2 years [1]. Owing to the rarity of SACC, clinical trials investigating systemic therapies are scarce and sample sizes are limited. In the last decade, several small phase II trials of chemotherapeutics/targeted therapies for locally recurrent/metastatic SACC have yielded modest success, with objective response rates of 0–20%, stable disease (SD) rates of 20–87%, and median overall survival of 6–27 months [2–14]. Based on these findings, Laurie et al. in a systematic review of studies of advanced SACC suggested that the preferred treatment option is clinical trial participation [15]. This is also the first of two options recommended by the National Comprehensive Cancer Network (NCCN) guidelines. The second is standard therapy, which includes chemotherapy for patients with a performance status (PS) of 0–2 and watchful waiting for slow-growing disease [16].

Molecular profiling (MP) of tumors may be used to identify potential targets for which there are available therapies. It involves the application of immunohistochemistry (IHC), fluorescent/chromogenic in situ hybridization (FISH/CISH), microarray analyses, sequencing, and reverse transcription polymerase chain reaction (RT-PCR). Treatment based on MP findings has proved successful in a pilot study of a variety of refractory cancers and a separate study of metastatic breast cancer [17, 18].

The objective of the present study was to evaluate a new MP-guided treatment paradigm in metastatic SACC in terms of feasibility and clinical outcomes.

## 2. Materials and Methods

### 2.1. Study Design

**Figure 1:**
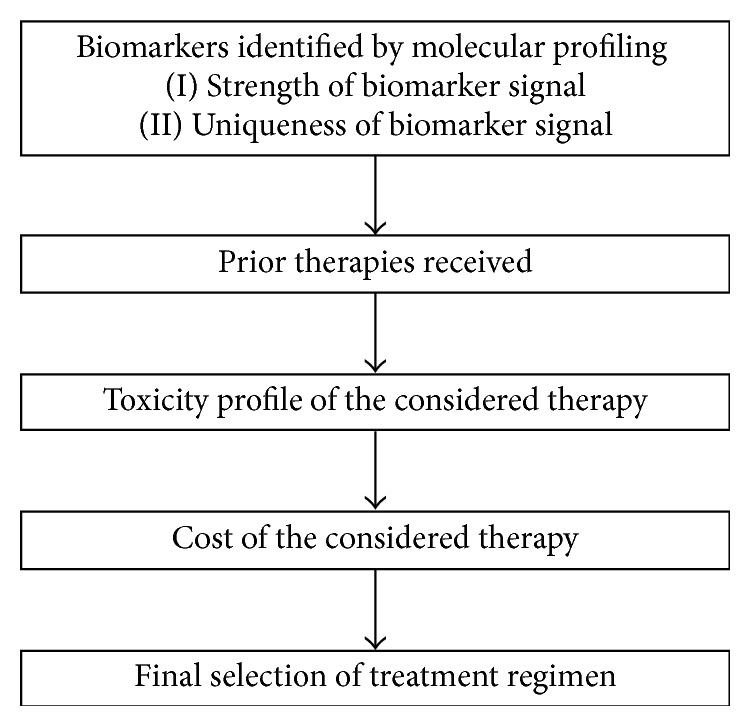
Considerations in choosing a treatment regimen after molecular profiling.

This single-center prospective cohort study included all patients with metastatic SACC who underwent MP at the Davidoff Center, Rabin Medical Center, from March 2010 to March 2013. The study was approved by the Institutional Review Board of Rabin Medical Center and all patients signed an informed consent. Therapy was selected on the basis of biomarkers identified by MP after considering multiple factors (Figure 1), including the strength and uniqueness of the biomarker signal, prior therapies received by the patient, the toxicity profile of the considered therapy, and the cost of the considered therapy. The regimens administered and the clinical outcomes were monitored prospectively; patients were followed until July 2014. Patient baseline characteristics and medical history were collected from the individual medical files.

The historical control cohort included all patients with metastatic SACC treated at the Davidoff Center from 1996 to 2009. Since the Davidoff Center has only recently become a referral center for head and neck malignancies, the historical cohort was considerably smaller than the study cohort. Patient data were collected from the medical files.

### 2.2. Molecular Profiling

MP was conducted on paraffin-embedded tissue taken from biopsy samples from the primary tumor or a metastatic lesion. Analyses were performed with the Caris Molecular Intelligence (CMI) tumor profiling service (Caris Life Sciences, Irving, TX) at the Caris Life Sciences Laboratories (Phoenix, AZ). They included IHC (for up to 18 targets), FISH/CISH to identify amplification in select genes, namely,* cMET *(the gene coding for the hepatocyte growth factor receptor protein; HGFR), epidermal growth factor receptor (*EGFR*), human epidermal growth factor receptor 2 (*HER2*), and topoisomerase II alpha (*TOP2A*). FISH was also used to identify rearrangement in the anaplastic lymphoma kinase (*ALK*) gene. Additionally, microarray analysis of over 80 targets and mutational analysis of* BRAF *(the gene coding for the serine/threonine-protein kinase B-raf protein),* KIT *(the gene coding for the mast/stem cell growth factor receptor Kit),* EGFR*,* KRAS *(the gene coding for the GTPase KRas protein), and* PIK3CA* (the gene coding for phosphatidylinositol 4,5-bisphosphate 3-kinase catalytic subunit alpha) were performed. In one patient, RT-PCR was used to determine gene expression.

IHC analysis was performed on formalin-fixed, paraffin-embedded tumor samples using commercially available detection kits, automated staining techniques (Benchmark XT, Ventana, Tusco, AZ, and AutostainerLink 48, Dako, Glostrup, Denmark), and commercially available antibodies-androgen receptor (AR), topoisomerases I and II*α* (TOPO1, TOPO*α*) (Leica Biosystems, Buffalo Grove, IL); estrogen receptor (ER), progesterone receptor (PgR); c-Met; HER2 (Ventana); tyrosine protein c-Kit receptor kinase (c-Kit), EGFR, and phosphatase and tensin homolog (PTEN) (Dako); O(6)-methylguanine-methyltransferase (MGMT); P-glycoprotein (PGP); thymidylate synthase (TS) (Invitrogen, Grand Island, NY); transducing-like enhancer of split 3 (TLE3) (Santa Cruz, Santa Cruz, CA); ribonucleotide reductase M1 (RRM1) (Protein Tech, Chicago, IL); Serum protein acidic and rich in cysteine (SPARC) (monoclonal, R&D Systems, Minneapolis, MN; polyclonal, Exalpha, Shirley, MA); and tubulin beta-3 chain (TUBB3) (Covance, Madison, WI). Results were categorized by defined cutoffs based on published evidence. Scoring system and cutoffs for all antibodies are provided in Supplementary Table 1, available online at http://dx.doi.org/10.1155/2015/614845.

FISH was used for evaluation of the* HER2 *(*HER2/CEP17* (chromosome 17 centromere) probe),* EGFR* (*EGFR/CEP7* probe),* TOPO2A* (*TOP2/CEP17* probe), and* cMET* (*c-MET/CEP7 *probe; Abbott Molecular/Vysis, Abbott Park, IL).* HER2/CEP17 *ratio >2.2 was considered amplified (based on guidelines from the College of American Pathology (CAP)/American Society of Clinical Oncology (ASCO) 2007).* EGFR* amplification was defined as* EGFR/CEP7* ratio ≥2 or ≥15* EGFR* copies per cell in ≥10% of analyzed cells.* TOPO2A *amplification was defined as* TOPO2A/CEP17* ratio ≥2.0 and* cMET* was considered amplified if ≥5* cMET* copies were detected on average.

Total RNA was extracted from tumor tissue and converted to cDNA. This cDNA sample was then subjected to a whole genome (24K) microarray analysis using Illumina cDNA-mediated annealing, selection, extension, and ligation process. The expression of a subset of 88 genes was then compared to a tissue-specific normal control and the relative expression ratios of these 88 target genes were determined as well as the statistical significance of the differential expression.

The types of analyses done and the specific targets assessed were determined by the amount of tissue sample available. If the amount was insufficient to perform all tests, the analyses were prioritized by the treating physician (based on various factors including prior therapies received, the likelihood of getting an actionable result for the particular marker, having access to the therapy that may be recommended as a result of testing, etc.). Another determining factor was the timeframe in which the MP was performed. CMI profiling evolved over the period of the study, with advances in methodologies and the introduction of published data about the relationship between certain biomarkers and response/resistance to therapy. An actionable target was defined as a target associated with clinical benefit from the matching therapies.

### 2.3. Clinical Outcomes

Response to treatment was based on Response Evaluation Criteria in Solid Tumors (RECIST 1.1). Disease control rate was defined as the proportion of patients with complete response (CR), partial response (PR), or SD. Response rate was defined as the proportion of patients with CR or PR. Disease control rate was defined as the proportion of patients with CR, PR, or SD. We also evaluated clinical benefit by monitoring PS, as defined by the Eastern Cooperative Oncology Group (ECOG) criteria [19], over time.

### 2.4. Statistical Analysis

Fisher's exact test was used to assess differences in treatment rates between the study cohort and the historical control cohort. Log-rank test was used to compare survival between the cohorts.

## 3. Results 

### 3.1. Patient Characteristics

Fourteen patients with metastatic SACC who were treated at the Davidoff Center between 2010 and 2013 and underwent MP were included in the study cohort. There were 9 male (64%) and 5 female (36%) patients of median age 57.5 years (range, 30–75) at diagnosis. Four patients (29%) had metastatic disease at diagnosis and 10 (71%) had localized disease at diagnosis which progressed to metastatic disease within a median of 25.3 months (range, 5.0–119.6) (Table 1). Three patients received treatment for metastatic disease prior to MP: paclitaxel, epirubicin, or cisplatin plus fluorouracil (5-FU) (1 each); none responded. At the time of progression to metastatic disease, 5 patients presented with a single metastatic site (lung, 4; brain, 1) and 9 with multiple metastatic sites (e.g., lung/liver, lung/spleen). At the time of MP, PS was 0 in 2 patients, 1 in 6 patients, and 2 in 6 patients.

The historical control cohort included 9 patients with metastatic SACC, of whom 4 (44%) were male. Median age was 58.0 years (range, 18–76). All were diagnosed with localized disease and underwent surgical resection. Progression to metastatic disease occurred within a median of 12.6 months (range, 3.0–71.0) from initial diagnosis. At the time of progression, 6 patients presented with a single metastatic site (all lung) and 3 with multiple metastatic sites (lung/liver, lung/bone, and lung/liver/bone) (Table 1).

### 3.2. Molecular Profiling Findings

The samples used for MP were derived from the primary tumor in 8 patients and metastasis in 6. IHC yielded reportable results for all samples. Microarray analysis was performed on 9 samples (successful in 6), FISH on 8 samples (successful in 5), and CISH on 2 samples (successful in 1). Molecular sequencing was performed on 4 samples and RT-PCR on one. Key MP findings for each patient in our study cohort are presented in Supplementary Table  2.

Overall, at least one (median, 5.5; range, 1–13) actionable target was identified for each patient (median, 5.5; range, 1–13). IHC identified a median of 3 actionable targets per patient (range, 1–7), with the most common being negative/low TS (9 of 12 evaluable patients), negative/low ERCC1 (6 of 12 evaluable patients), and high TOPO1 (6 of 13 evaluable patients) (Table 2). It should be noted that the usefulness of ERCC1 as a biomarker has recently been questioned [20], and, consequently, ERCC1 testing is now available only upon request. None of the patients was HER2-positive by IHC, FISH, or CISH. Targets associated with an endocrine-therapy benefit included positive AR and positive ER/PgR (2 of 14 evaluable patients each) (Table 2). No* EGFR* gene amplification (4 evaluable patients) or* ALK* rearrangement (3 evaluable patients) was observed by FISH. Microarray analysis identified at least 2 actionable targets per patient (median, 8; range, 2–10) in 6 evaluable patients, including* KIT* overexpression and topoisomerase II beta (*TOP2B*) overexpression in 4 patients, each. RT-PCR identified 8 actionable targets in 1 evaluable patient, and sequencing analyses, conducted in 4 patients, revealed wild-type phenotypes for all evaluated genes.

### 3.3. Treatments and Clinical Outcomes

**Figure 2:**
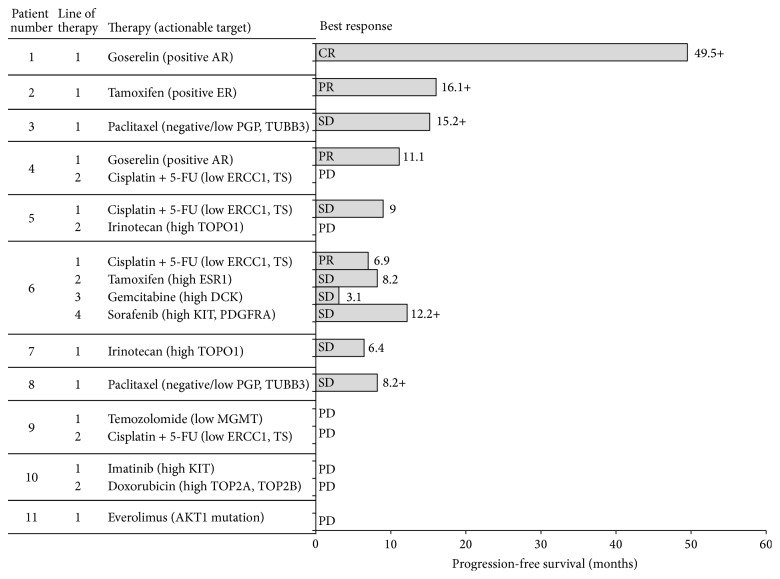
Treatment and progression-free survival in study cohort patients treated with MP-guided therapy (*n* = 11). 5-FU: fluorouracil; AR: androgen receptor; CR: complete response; DCK: deoxycytidine kinase; ESR1: estrogen receptor 1; ER: estrogen receptor; ERCC1: excision repair cross-complementation 1; MGMT: O-6-methylguanine-DNA methyltransferase; PDGFRA: platelet-derived growth factor receptor alpha; PGP: P-glycoprotein; PR: partial response; SD: stable disease; TOP2A/B: topoisomerase IIA/B; TOPO1: topoisomerase I; TS: thymidylate synthase; TUBB3: tubulin, beta 3 c1ass III.

The treatments associated with the identified targets included agents that are routinely used in the metastatic SACC setting (i.e., cisplatin, 5-FU) and therapies that are rarely used in this setting (i.e., temozolomide, endocrine therapies) (Figure 2).

Of the 14 patients in the study cohort, 2 died before initiation of treatment and 1 opted not to be treated. Overall, 11 of 14 patients (79%) received MP-guided therapy. These included 1 patient whose sample underwent 2 panels of tests, CMI and sequencing, and whose therapy was selected based on the sequencing-identified mutation. In total, 18 MP-guided regimens were administered: 6 patients received 1 line of treatment each, 4 received 2 lines, and 1 received 4 lines.

**Figure 3:**
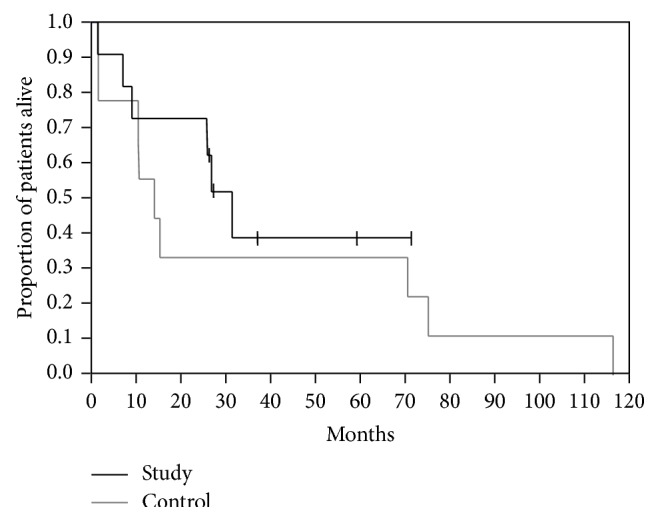
Kaplan-Meier survival curves (from diagnosis of metastatic disease) for study patients who received MP-guided therapy (*n* = 11) and historical control patients (*n* = 9). Tick marks indicate censored observations.

Median duration of follow-up from initiation of MP-guided therapy was 11.8 months. In the first line of treatment, 1 of the 11 patients (9.1%) achieved CR which continued for >4 years, 3 (27.3%) achieved PR, and 4 (36.9%) had SD, for a response rate of 36% and a disease control rate of 73%. Median progression-free survival for first-line MP-guided therapy was 8.2 months; 1 patient had a progression-free survival of 49.5+ months (Figure 2). Median overall survival with metastatic disease was 31.3 months (range 1.4–71.1+) (Figure 3). In the 4 patients with CR/PR, the PS improved by 1 or 2 categories to 0. In 3 of the 5 patients with SD, PS improved by 1 category.

Of the historical control patients who attended our center before MP was available, only 2 (22%) were treated in the metastatic setting, as opposed to 11 (79%) in the study cohort (*P* = 0.013; Fisher's exact test). One of them received cisplatin/doxorubicin/cyclophosphamide for lung metastases and achieved a PR for 24 months, followed by disease progression. This patient also underwent radiation therapy and participated in 2 clinical trials, with no response. She died approximately 9 years after diagnosis of the metastatic disease. The second patient received cisplatin/doxorubicin/5-FU for liver and bone metastases with no response and died 47 days after progression to metastatic disease. The other 7 patients were not treated. Median overall survival with metastatic disease was 14.0 months (range, 1.5–116). It was shorter than for the study cohort (31.3 months), but the difference was not statistically significant, probably because of the small sample size (*P* = 0.45, log-rank test; Figure 3).

## 4. Discussion

Herein, we report a single-institution experience in implementing a targeted comprehensive MP testing in 14 patients with advanced SACC over a period of 4.5 years. MP findings were feasible, and the majority of patients (79%) were treated based on the molecular profile of their tumor, with some gaining substantial clinical benefit.

IHC analysis was successful in all samples, unlike microarray and FISH analyses which were not possible for all samples due to technical failure of the testing. In samples where microarray/FISH testing failed, high quality expression data could not be obtained despite multiple attempts. The ability to detect expression changes is directly proportional to the amount of tumor nuclei present in the patient sample. In comparison to microarray/FISH analyses, IHC may be performed on much smaller samples, needing a minimum of 100 tumor cells to be present; therefore, it could be more readily performed in samples with low tumor yield.

To study the efficacy of this approach, clinical outcomes were compared to historical control patients treated in the same institution. The results demonstrate that MP-guided therapy is feasible in this setting and leads to good clinical outcomes in patients who might otherwise not have further treatment options. The significantly greater number of patients in the study cohort who received therapy compared to the historical control cohort suggests that this approach broadens the armamentarium of available treatments for metastatic SACC and provides clinicians with the opportunity to offer additional therapy when sought by patients with disease progression or poor tolerability. We found that MP-guided therapy was associated with a disease control rate of 73% (CR 9.1%, PR 27.3%, and SD 36.4%) and an overall improvement in PS in patients with CR/PR/SD. The small number of patients restricts our ability to draw definitive conclusions. Nevertheless, the similar disease control rate and better response rate in the present study compared to phase II studies of advanced SACC published in the last decade [2–14] together suggest that MP-guided therapy may improve clinical outcomes. Notably, in this hard-to-treat patient population, SD is a desirable objective, as it may lead to improvements in disease-related symptoms, quality-of-life, and survival [21]. Furthermore, the overall survival of the study cohort was longer than that of the historical controls (albeit not significantly owing to the small sample sizes).

Treatment based on MP could be particularly beneficial for cancers with molecular heterogeneity and for low-prevalence cancers, which are hardly studied in clinical trials. Moreover, given that MP takes up to two weeks, this approach may be more suitable for relatively slowly progressing diseases such as SACC, and, even in these cases, it should be reserved for patients whose condition is relatively stable.

Previous studies have demonstrated the feasibility and potential clinical benefit of the MP-guided approach in a variety of refractory cancers and in metastatic breast cancer where it resulted in a ≥30% longer progression-free survival than for the last (pre-MP) regimen on which the patients progressed in 27% and 52% of cases, respectively [17, 18]. Although the progression-free survival ratio endpoint was inappropriate for our study, as most of the patients did not receive pre-MP chemotherapy for metastatic disease, our findings are compatible with these earlier studies and support the need for further investigations of MP-guided therapy.

MP-guided therapy is inherently limited by its ability to assess only tumor-based parameters (molecular characteristics of tumor cells and adjacent cells), without consideration of effects related to systemic drug distribution and some immunological aspects of cancer therapy [22, 23].

The study is limited by its nonrandomized design and inclusion of only 14 patients, although a sample this size may be adequate for such a rare disease. Furthermore, the Davidoff Center specializes in head and neck malignancies and, recently, patients from all over Israel have begun to be referred there. Consequently, our study cohort may have included a higher proportion of hard-to-treat patients compared to the general patient population.

In conclusion, our study suggests that MP-guided therapy is feasible for metastatic SACC and leads to substantial clinical benefit in some patients. This approach could be crucial for improving clinical outcomes as it facilitates personalized treatment. This study constitutes a proof of concept for the feasibility of this approach in other malignancies characterized by lack of standardized treatment, rarity of the tumor, and relatively slow disease progression.

## Supplementary Material

The supplementary material provides details on the scoring system and cutoffs used for the immunohistochemistry analyses (Supplementary Table 1), as well as key molecular-profiling findings for each of the 14 patients in our study cohort (Supplementary Table 2).

## Figures and Tables

**Table 1:** Patient and tumor characteristics.

Characteristic	Study cohort *N* = 14	Control cohort *N* = 9
Gender, *n* (%)		
Male	9 (64.3)	4 (44.4)
Female	5 (35.7)	5 (55.6)
Age at diagnosis, years		
Median (range)	57.5 (30–75)	58 (18–76)
Stage at diagnosis		
Localized	10 (71.4)	9 (100)
Metastasized	4 (28.6)	0 (0)
Prior surgery		
Yes	9 (64.3)	9 (100)
No	5 (35.7)	0 (0)
Prior radiation		
Yes	9 (64.3)	8 (88.9)
No	5 (35.7)	1 (11.1)
Time to progression to metastatic disease, months		
Median (range)	25.3 (5.0–119.6)^*∗*^	12.6 (3.0–71.0)
At progression to metastatic disease^†^		
Patients with a single metastatic site, *n* (%)	5 (35.7)	6 (66.7)
Patients with multiple metastatic sites, *n* (%)	9 (64.3)	3 (33.3)
Chemotherapy for metastatic disease		
Yes	3 (21.4)^‡^	2 (22.2)
No	11 (78.6)	7 (77.8)

^*∗*^For patients presenting with localized disease.

^†^At presentation of metastatic disease.

^‡^Prior to molecular profiling.

**Table 2:** Actionable targets identified by molecular profiling in the study cohort.

Target	Number of patients out of total evaluable patients (*n*/*N*)	Frequency, %
Identified by IHC		
Negative/low TS	9/12	75
Negative/low ERCC1	6/12	50
High TOPO1	6/13	46
High SPARC^*∗*^	4/11	36
Low MGMT	3/14	21
High TOP2A	2/11	18
Positive AR	2/14	14
Positive ER/PgR	2/14	14
Positive HER2	0/14	0
Identified by microarray analysis		
KIT overexpression	4/6	67
TOP2B overexpression	4/6	67
PDGFRA overexpression	3/6	50
PDGFRB overexpression	3/6	50
TOP2A overexpression	3/6	50
TYMS overexpression	2/6	33
VDR overexpression	2/6	33
ESR1 overexpression	2/6	33
SPARC overexpression	2/6	33
MGMT underexpression	2/6	33

^*∗*^SPARC was considered high if either of the SPARC assays (using monoclonal or polyclonal anti-SPARC antibodies) was positive.

AR: androgen receptor; ER: estrogen receptor; ERCC1: excision repair cross-complementation 1; ESR1: estrogen receptor 1; HER2: human epidermal growth factor receptor 2; IHC: immunohistochemistry; MGMT: O-6-methylguanine-DNA methyltransferase; PDGFRA/B: platelet-derived growth factor receptor alpha/beta; PgR: progesterone receptor; SPARC: secreted protein acidic and rich in cysteine; TOPO1: topoisomerase I; TOP2A/B: topoisomerase IIA/B; TS/TYMS: thymidylate synthase; VDR: vitamin D receptor.

## References

[1] Spiro R. H. (1997). Distant metastasis in adenoid cystic carcinoma of salivary origin. *American Journal of Surgery*.

[2] Chau N. G., Hotte S. J., Chen E. X. (2012). A phase II study of sunitinib in recurrent and/or metastatic adenoid cystic carcinoma (ACC) of the salivary glands: current progress and challenges in evaluating molecularly targeted agents in ACC. *Annals of Oncology*.

[3] Glisson B. S., Blumenschein G., Francisco M., Erasmus J., Zinner R., Kies M. (2005). Phase II trial of gefitinib in patients with incurable salivary gland cancer. *Journal of Clinical Oncology*.

[4] Argiris A., Goldwasser M. A., Burtness B. (2006). A phase II trial of PS-341 (bortezomib) followed by the addition of doxorubicin at progression in incurable adenoid cystic carcinoma of the head and neck: an eastern cooperative oncology group study. *Proceedings of the American Society of Clinical Oncology*.

[5] Agulnik M., Cohen E. W. E., Cohen R. B. (2007). Phase II study of lapatinib in recurrent or metastatic epidermal growth factor receptor and/or erbB2 expressing adenoid cystic carcinoma and non-adenoid cystic carcinoma malignant tumors of the salivary glands. *Journal of Clinical Oncology*.

[6] Locati L. D., Bossi P., Perrone F. (2009). Cetuximab in recurrent and/or metastatic salivary gland carcinomas: a phase II study. *Oral Oncology*.

[7] Guigay J. M., Bidault F., Temam S. (2007). Antitumor activity of imatinib in progressive, highly expressing KIT adenoid cystic carcinoma of the salivary glands: a phase II study. *Journal of Clinical Oncology—ASCO Annual Meeting Proceedings*.

[8] Lin C. H., Yen R. F., Jeng Y. M., Tzen C. Y., Hsu C., Hong R. L. (2005). Unexpected rapid progression of metastatic adenoid cystic carcinoma during treatment with imatinib mesylate. *Head and Neck*.

[9] Pfeffer M. R., Talmi Y., Catane R., Symon Z., Yosepovitch A., Levitt M. (2007). A phase II study of Imatinib for advanced adenoid cystic carcinoma of head and neck salivary glands. *Oral Oncology*.

[10] Hotte S. J., Winquist E. W., Lamont E. (2005). Imatinib mesylate in patients with adenoid cystic cancers of the salivary glands expressing c-kit: a Princess Margaret Hospital Phase II Consortium Study. *Journal of Clinical Oncology*.

[11] Laurie S. A., Siu L. L., Winquist E. (2010). A phase 2 study of platinum and gemcitabine in patients with advanced salivary gland cancer: a trial of the NCIC clinical trials group. *Cancer*.

[12] Ross P. J., Teoh E. M., A'Hern R. P. (2009). Epirubicin, cisplatin and protracted venous infusion 5-Fluorouracil chemotherapy for advanced salivary adenoid cystic carcinoma. *Clinical Oncology*.

[13] Gilbert J., Li Y., Pinto H. A. (2006). Phase II trial of taxol in salivary gland malignancies (E1394): a trial of the Eastern Cooperative Oncology Group. *Head and Neck*.

[14] van Herpen C. M. L., Locati L. D., Buter J. (2008). Phase II study on gemcitabine in recurrent and/or metastatic adenoid cystic carcinoma of the head and neck (EORTC 24982). *European Journal of Cancer*.

[15] Laurie S. A., Ho A. L., Fury M. G., Sherman E., Pfister D. G. (2011). Systemic therapy in the management of metastatic or locally recurrent adenoid cystic carcinoma of the salivary glands: a systematic review. *The Lancet Oncology*.

[16] http://www.nccn.org/professionals/physician_gls/pdf/head-and-neck.pdf.

[17] Von Hoff D. D., Stephenson J. J., Rosen P. (2010). Pilot study using molecular profiling of patients' tumors to find potential targets and select treatments for their refractory cancers. *Journal of Clinical Oncology*.

[18] Jameson G. S., Petricoin E., Sachdev J. C. (2013). A pilot study utilizing molecular profiling to find potential targets and select individualized treatments for patients with metastatic breast cancer. *Journal of Clinical Oncology*.

[19] Oken M. M., Creech R. H., Tormey D. C. (1982). Toxicity and response criteria of the Eastern Cooperative Oncology Group. *American Journal of Clinical Oncology*.

[20] Friboulet L., Olaussen K. A., Pignon J.-P. (2013). ERCC1 isoform expression and DNA repair in non-small-cell lung cancer. *The New England Journal of Medicine*.

[21] Kelly K. (2003). The benefits of achieving stable disease in advanced lung cancer. *Oncology*.

[22] Zhou S.-F., Di Y. M., Chan E. (2008). Clinical pharmacogenetics and potential application in personalized medicine. *Current Drug Metabolism*.

[23] Zitvogel L., Apetoh L., Ghiringhelli F., Kroemer G. (2008). Immunological aspects of cancer chemotherapy. *Nature Reviews Immunology*.

