# Supplementary material for: Metastatic Salivary Gland Tumors: A Single-Center Study Demonstrating the Feasibility and Potential Clinical Benefit of Molecular-Profiling-Guided Therapy
Source: Biomed Res Int. 2015 Sep 13;2015:614845. doi: 10.1155/2015/614845 (PMC4584046; doi:10.1155/2015/614845)
Supplement: Supplementary file 1 — The supplementary material provides details on the scoring system and cutoffs used for the immunohistochemistry analyses (Supplementary Table 1), as well as key molecular-profiling findings for each of the 14 patients in our study cohort (Supplementary Table 2). [file 614845.f1.pdf]

Supplementary Tables

Supplementary Table 1: Thresholds used for immunohistochemistry analyses

Marker	Threshold (marker intensity/percentage)
Androgen Receptor (AR)	= 0+ or <10% or ≥1+ and ≥10%
c-KIT (CD117)	=0+ and =100% or ≥2+ and ≥30%
cMET	<50% or <2+ or ≥2+ and ≥50%
Estrogen Receptor (ER)	=0+ or <10% or ≥1+ and ≥10%
Progesterone Receptor (PR)	=0+ or <10% or ≥1+ and ≥10%
Epidermal Growth Factor Receptor (EGFR)	2+ and ≥10%
Human Epidermal Growth Factor Receptor 2 (HER2)	≤1+ or =2+ and ≤10% or ≥3+ and >10%
O(6)-methylguanine-methyltransferase (MGMT)	=0+ or ≤35% or ≥1+ and >35%
P-Glycoprotein (PGP)	=0+ or <10% or ≥1+ and ≥10%
Phophatase and Tensin Homolog (PTEN)	=0+ or ≤50% or ≥1+ and >50%
Ribonucleotide Reductase M1 (RRM1)	=0+ or <50% or <2+ or ≥2+ and ≥50%
SPARC (Osteonectin)	<30% or <2+ or ≥2+ and ≥30%
Transducin-Like Enhancer of Split 3 (TLE3)	<30% or <2+ or ≥2+ and ≥30%
Topoisomerase 2 Alpha (TOPO2A)	=0+ or <10% or ≥1+ and ≥10%
Topoisomerase 1 (TOPO1)	=0+ or <30% or <2+ or ≥2+ and ≥30%
Thymidylate Synthase (TS)	=0+ or ≤3+ and <10% or ≥1+ and ≥10%
Tubulin β-3 Chain (TUBB3)	<30% or <2+ or ≥2+ and ≥30%

Supplementary Table 2: Key MP-findings for 14 cases of metastatic salivary gland ACC.

	IHC (key targets)								FISH	Microarray[a]	Sequencing
**Patient No.**	**TS**	**ERCC1**	**TOPO1**	**SPARC[b]**	**MGMT**	**TOP2A**	**ER/PgR**	**AR**	**Targets successfully measured by FISH**	**Number of significant targets identified**	**Mutations identified by sequencing**
1	**Neg**	High	Low	Low	Int.	Low	Neg	**Pos**	Analysis failed	**3**	Analysis not performed
2	**Neg**	**Low**	Low	NA	**Low**	Low	**Pos**	Neg	Analysis not performed	Analysis not performed	Analysis not performed
3[c]	**Low**	High	Low	**High**	High	NA	Neg	Neg	TOP2A, neg; cMET, neg	Analysis not performed	Analysis failed
4	**Low**	**Low**	Low	Low	High	Low	Neg	**Pos**	Analysis failed	Analysis failed	Analysis not performed
5	**Low**	**Low**	**High**	Low	High	Low	Neg	Neg	Analysis failed	**10**	Analysis not performed
6	**Low**	**Low**	Low	**High**	Int.	Low	Neg	Neg	EGFR, neg; HER2, neg; ALK, no rearrangement	**9**	**BRAF: w.t; EGFR and PIK3CA sequencing: failed**
7	**Low**	High	**High**	Low	High	Low	Neg	Neg	Analysis not performed	Analysis failed	Analysis not performed
8[c]	Neg	NA	**High**	**High**	High	Low	Neg	Neg	Analysis not performed	Analysis not performed	Analysis failed
9	**Low**	**Low**	Low	Low	**Low**	**High**	**Pos**	Int.	EGFR, neg; ALK, no rearrangement	**7**	**BRAF, C-KIT, EGFR, KRAS: all w.t.**
10	High	**Low**	Low	Low	High	**High**	Neg	Neg	EGFR, neg; HER2, neg; ALK, no rearrangement	**8**	**BRAF, EGFR, PIK3CA: all w.t.**
11	NA	NA	NA	**High**	**Low**	NA	Neg	Neg	Analysis not performed	Analysis not performed	Analysis not performed[d]
12	**Neg**	High	**High**	Low	Int.	Low	Neg	Neg	Analysis not performed	**2**	Analysis not performed
13	Int.	Int.	**High**	NA	High	Low	Neg[e]	NA	Analysis not performed	Analysis not performed	**C-KIT: w.t; EGFR sequencing: failed**
14	NA	High	**High**	NA	High	NA	Neg	Neg	EGFR, neg; HER2, neg	Analysis failed	Analysis not performed
**No. of patients with actionable[f] target/no. of evaluable patients (%)**	**9/12 (75%)**	**6/12 (50%)**	**6/13 (46%)**	**4/11 (36%)**	**3/14 (21%)**	**2/11 (18%)**	**2/14 (14%)**	**2/14 (14%)**	**NA**	**NA**	**NA**

Grey cells with bold font represent findings associated with potential clinical benefit for a particular treatment regimen. None of the 12 patients whose HER2 levels were tested by IHC was found to be HER2 positive.

ACC, adenoid cystic carcinomas; AR, androgen receptor; ASNS, asparagine synthetase; CMI, Caris Molecular Intelligence; DCK, deoxycytidine kinase; EGFR, epidermal growth factor receptor; EPHA2, erythropoietin-producing hepatocellular receptor tyrosine kinase class A2; ER, estrogen receptor; ERCC, excision repair cross-complementation; ESR1, estrogen receptor 1; FISH, fluorescence in situ hybridization; HER2, human epidermal growth factor receptor 2; IHC, immunohistochemistry; Int, intermediate; MGMT, O-6-methylguanine-DNA methyltransferase; MP, molecular profiling; NA, not available; Neg, negative; 1; PDGFRA, platelet-derived growth factor receptor alpha; PGP,P-glycoprotein; PgR, progesterone receptor; Pos, positive; RRM2B, ribonucleotide reductase M2 B; SPARC, secreted protein acidic and rich in cysteine; SSTR2, somatostatin receptor 2; TOPO1/TOP1, topoisomerase I; TOP2A/B, topoisomerase II alpha/beta; TS, thymidylate synthase; TUBB3, tubulin, beta 3 class III; VDR, vitamin D receptor; VEGFR2, vascular endothelial growth factor receptor; w.t., wild type.

[a]Microarray analysis evaluated 80 targets.
[b]The IHC analysis was performed twice (using monoclonal and polyclonal anti SPARC antibodies).
[c]These patients had negative/low PGPand TUBB3 by IHC
[d]This patient underwent 2 sets of MP, CMI and sequencing (not through CMI) and was found to have AKT1 mutation
[e]This patient had PR negative tumor, ER levels were not measured.
[f]An actionable target is defined as an MP finding associated with a potential clinical benefit for a particular treatment regimen.
